# Pesticide application has little influence on coding and non-coding gene expressions in rice

**DOI:** 10.1186/s12864-019-6381-y

**Published:** 2019-12-23

**Authors:** Sajid Muhammad, Jingai Tan, Pingchuan Deng, Tingting Li, Haohua He, Jianmin Bian, Liang Wu

**Affiliations:** 1 College of Agriculture and Biotechnology, Zhejiang University, Hangzhou, 310058 China; 2 Key Laboratory of Crop Physiology, Ecology and Genetic Breeding, Ministry of Education, Jiangxi Agricultural University, Nanchang, 330045 China

**Keywords:** Rice, Pesticide, Long non-coding RNAs, Alternative splicing, Abiotic stress

## Abstract

**Background:**

Agricultural insects are one of the major threats to crop yield. It is a known fact that pesticide application is an extensive approach to eliminate insect pests, and has severe adverse effects on environment and ecosystem; however, there is lack of knowledge whether it could influence the physiology and metabolic processes in plants.

**Results:**

Here, we systemically analyzed the transcriptomic changes in rice after a spray of two commercial pesticides, Abamectin (ABM) and Thiamethoxam (TXM). We found only a limited number of genes (0.91%) and (1.24%) were altered by ABM and TXM respectively, indicating that these pesticides cannot dramatically affect the performance of rice. Nevertheless, we characterized 1140 Differentially Expressed Genes (DEGs) interacting with 105 long non-coding RNAs (lncRNAs) that can be impacted by the two pesticides, suggesting their certain involvement in response to farm chemicals. Moreover, we detected 274 alternative splicing (AS) alterations accompanied by host genes expressions, elucidating a potential role of AS in control of gene transcription during insecticide spraying. Finally, we identified 488 transposons that were significantly changed with pesticides treatment, leading to a variation in adjacent coding or non-coding transcripts.

**Conclusion:**

Altogether, our results provide valuable insights into pest management through appropriate timing and balanced mixture, these pesticides have no harmful effects on crop physiology over sustainable application of field drugs.

## Background

Insect pests (IPs) are the most prominent threats in achieving global food demands of a rapidly growing population. IPs affect the latent yield of all agricultural crops either directly or indirectly. Direct damage may include deformations or necrosis of plant tissues or organs, fouling and dispersion of plant pathogens, while the loss of harvest quality and increase in the cost of crop production may involve indirect damage [1, 2].

Owing to the severity of agricultural insects’ problem, it has become a great challenge to use sustainable measures to control IPs that could affect crop yield. Various control strategies including mechanical, biological, cultural, transgenic and chemical have been followed by farmers to manage IPs since past. Modern biotechnology and genetic engineering led to the development of Genetically Modified Organisms (GMOs) of plants, animals or microorganisms, whose genetic material has been altered using genetic engineering techniques. However, GMOs are still controversial and raising some concerns over food safety in long terms [3].

The effective management of IPs mainly depends on chemical control methods so far, such as the application of pesticides, which is the quickest and most hard-hitting control method [4]. Many pesticides are also involved in the enhancement of agricultural production through the expurgation of soil-borne pathogens. In paddy fields, nearly 15% of the total plant protection, chemicals are used for crop production [5]. Among them, Abamectin (ABM) and Thiamethoxam (TXM) are the most impelling systematic pesticides widely used for rice, soybean, sunflower, cotton and potato seed treatments as well as in fields nowadays [6, 7].

ABM, the pesticide used to treat IPs, naturally generated as fermentation products by *Streptomyces avermitilis*, a soil actinomycete [8]. ABM blocks nerve and muscle cells of the insects mostly by enhancing the effects of glutamate at the invertebrate-specific glutamate-gated chloride channel with minor impact on gamma-aminobutyric acid receptors [9–11]. This barricade causes an influx of chloride ions into the cells, leading to a hyper polarization and subsequent paralysis of invertebrate neuromuscular systems, while comparable doses are not toxic for mammals, as they do not possess glutamate-gated chloride channels [12].

TXM is a neonicotinoid that can be absorbed quickly by plants and transported to all of its tissues, including pollens where it acts to deter insect-feeding. This compound interferes with nicotinic acetylcholine receptors in the central nervous system of insects, and eventually paralyzes their muscular movements [13]. TXM has been widely used because it controls a broad range of IPs while possessing relatively low mammalian toxicity [14, 15].

Although it is clear that pesticides can kill crop insects, it is still elusive whether they can affect plant growth and physiological performance [16]. Generally, we could not see an obvious alteration of plant development after spraying a commercial pesticide, but this doesn’t mean that pesticide can’t influence the endogenous metabolic processes of crops, which may indirectly bring about human health issues. Thus, evaluating the effects of pesticides on crop physiology are crucial for IP control programs.

Rice (*Oryza sativa L.*) is a major staple cereal in the world, providing essential caloric requirements for more than half of the world’s population. To satisfy the gradually increasing food demands for a rapidly growing population, rice yields need to be increased up to 40% by 2030 [17]. Meanwhile, many rice insects including brown plant hopper, leaf roller, and stem borer result in a major threat to rice production. To date, diverse insecticides have been used to suppress rice pests in an open field; among them, the application of ABM and TXM is the major solution for killing masticatory and sucking IPs. However, apart from crop safety, it is unknown whether plant physiology is compromised by the two pesticides, thereby triggering an interesting question to be addressed.

RNA sequencing (RNA-Seq) is a powerful tool to examine the continuously changing cellular transcriptome, thereby facilitates the ability to know potential physiological changes under distinct conditions. In this study, we conducted RNA-Seq analysis to determine rice dynamic performances after ABM and TXM spray through characterization of Differentially Expressed Genes (DEGs), Differentially Expressed Alternatively Spliced RNAs (DE AS), Differentially Expressed Long Non-Coding RNAs (DE lncRNAs) and Differentially Expressed Transposable elements (DE TEs). We found that a limited number of these coding and non-coding transcripts can be overlapped or exclusively changed along with the application of two different pesticides. These results provide valuable insights into the proper usage of pesticides against masticatory and sucking IPs in crops.

## Results

### Identification of differentially expressed genes (DEGs) under Abamectin (ABM) treated rice

**Fig. 1:**
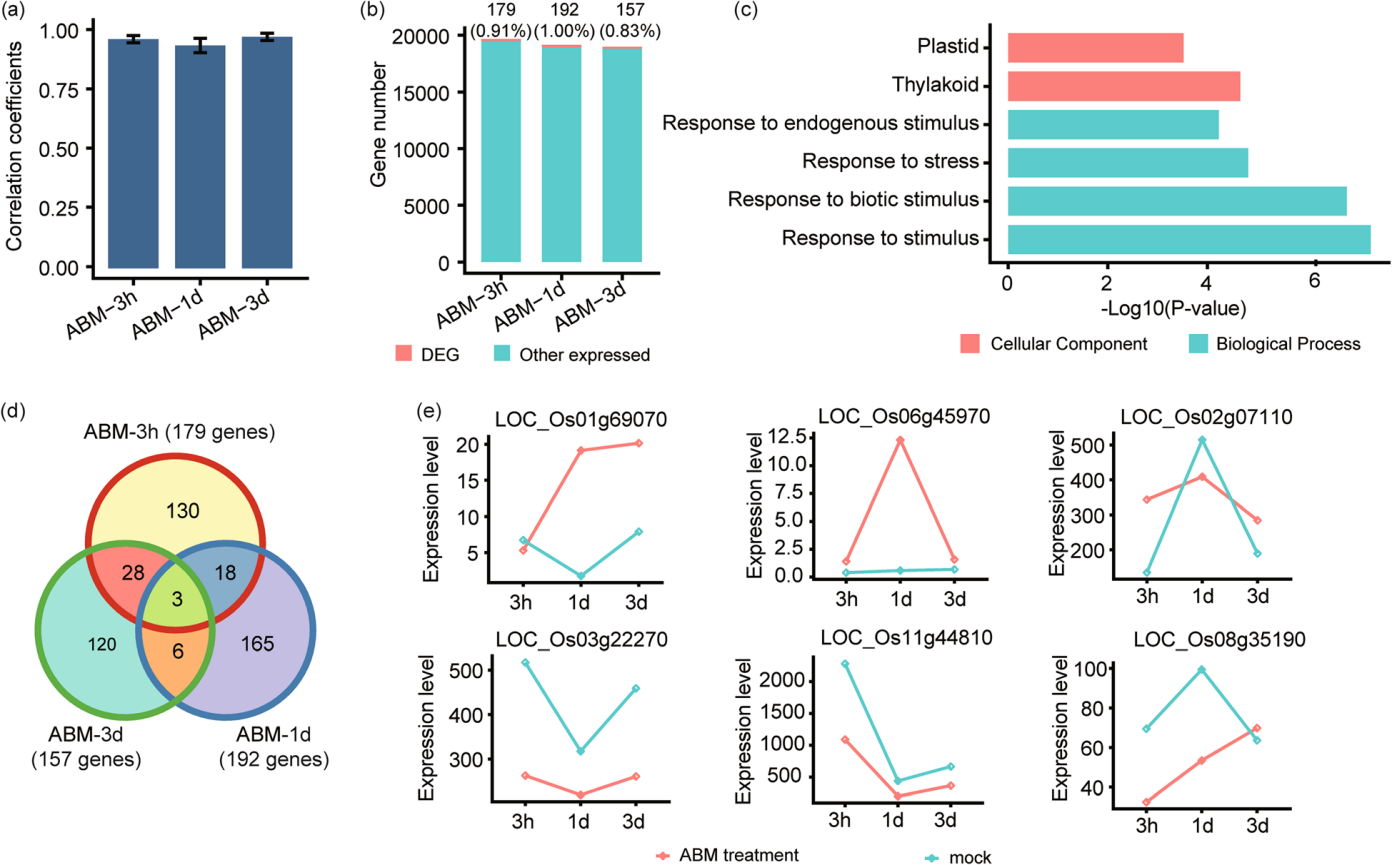
Expression pattern and functional analysis of differentially expressed genes (DEGs) in rice inoculated with Abamectin (ABM). **a** Bar graphs depict correlation co-efficients (R) of ABM under three treatments, i.e., 3 h, 1d, and 3d. The y-axis represents correlation co-efficient of treatments, and x-axis shows pesticide treatments. **b** Proportionate percentages of DEGs to other expressed genes, red color in the bar graph shows the proportion of DEGs to other expressed genes illustrated in blue color. **c** Overview of Gene Ontology analysis of all DEGs under ABM application. The x-axis represents the negative log of the *P*-value, and y-axis shows GO terms. **d** Venn diagram describing total, unique and overlaps among DEGs after three treatments of ABM, the number of shared DEGs are specified in circles. **e** Expressions of selected DEGs based on high throughput sequencing, under control and ABM, treated plants. The y-axis is the FPKM (Fragments Per Kilobase of exon per Million reads) values for each gene and x-axis represents treatments of ABM. First two genes are the typical examples of induced genes under ABM compared with control, while others are examples for low expressed genes under ABM treatments

Pesticides can kill crop IPs, but their influence on different biological and physiological processes are still elusive. To investigate rice transcriptome in response to pesticides, we carried out RNA-Seq and measured FPKM values of genes under ABM treatment. A total of 470 DEGs were annotated in rice under ABM treatments. Correlation coefficients (R) of all the treatments were near to 1, showing a high repetition of the experiment in terms of data analysis, expression and sequence coverage (Fig. 1a, Additional file 1). To determine reliability in the transcriptome gene expressions (GE) profiles in ABM treatments, we randomly checked the expression patterns of six DEGs using RT-qPCR. Expression patterns of all the examined genes were similar to RNA-seq data, indicating the credibility of our transcriptome dataset for gene exploration (Additional file 2). Hence, it would be reliable to find out the influence of pesticide by our RNA-seq dataset. We compared DEGs with other expressed genes in relation to their percentages, and got the highest number of 192 DEGs (1.00%) under 1 day (1d) ABM treated plants, followed by 179 (0.91%) DEGs under 3 h (3 h) treatment, and 157 (0.83%) DEGs under 3 days (3d) treatment. These results indicated that DEGs were less in number compared to non-altered genes, and further implicated that the insecticide has a little grasp on GE level, mostly impacting 1d treated plants (Fig. 1b).

To further investigate the potential functions of DEGs, we identified their localization into different cellular components or biological processes under GO terms (Fig. 1c). Besides specifically expressed DEGs under three treatments of ABM, there were still some overlaps among DEGs per time point (Fig. 1d, Additional file 3), e.g., 3 h and 1d treatments shared 18 DEGs, six between 1d and 3d treatments, while 28 shared DEGs were recorded among 3d and 3 h. Apart from this, we also have three co-expressed DEGs shared by all treatments (Fig. 1d, Additional file 3). To further pursue dynamic changes in DEGs, we measured the FPKM values of genes under different time treatments of ABM. We observed two genes, *Os01g69070* and *Os06g45970*, which are involved in Auxin response [18], were particularly induced, suggesting a potential alteration in auxin signaling by ABM treatments (Fig. 1e).

### Identification of DEGs under Thiamethoxam (TXM) applied rice

Since we didn’t observe a severe alteration in GE after spraying ABM, we attempted to select another commercial pesticide to check whether it can lead to a significant change in rice transcriptomes. Due to the widespread use of TXM, it would be worthwhile to study its influence on the endogenous metabolic processes in plants.

**Fig. 2:**
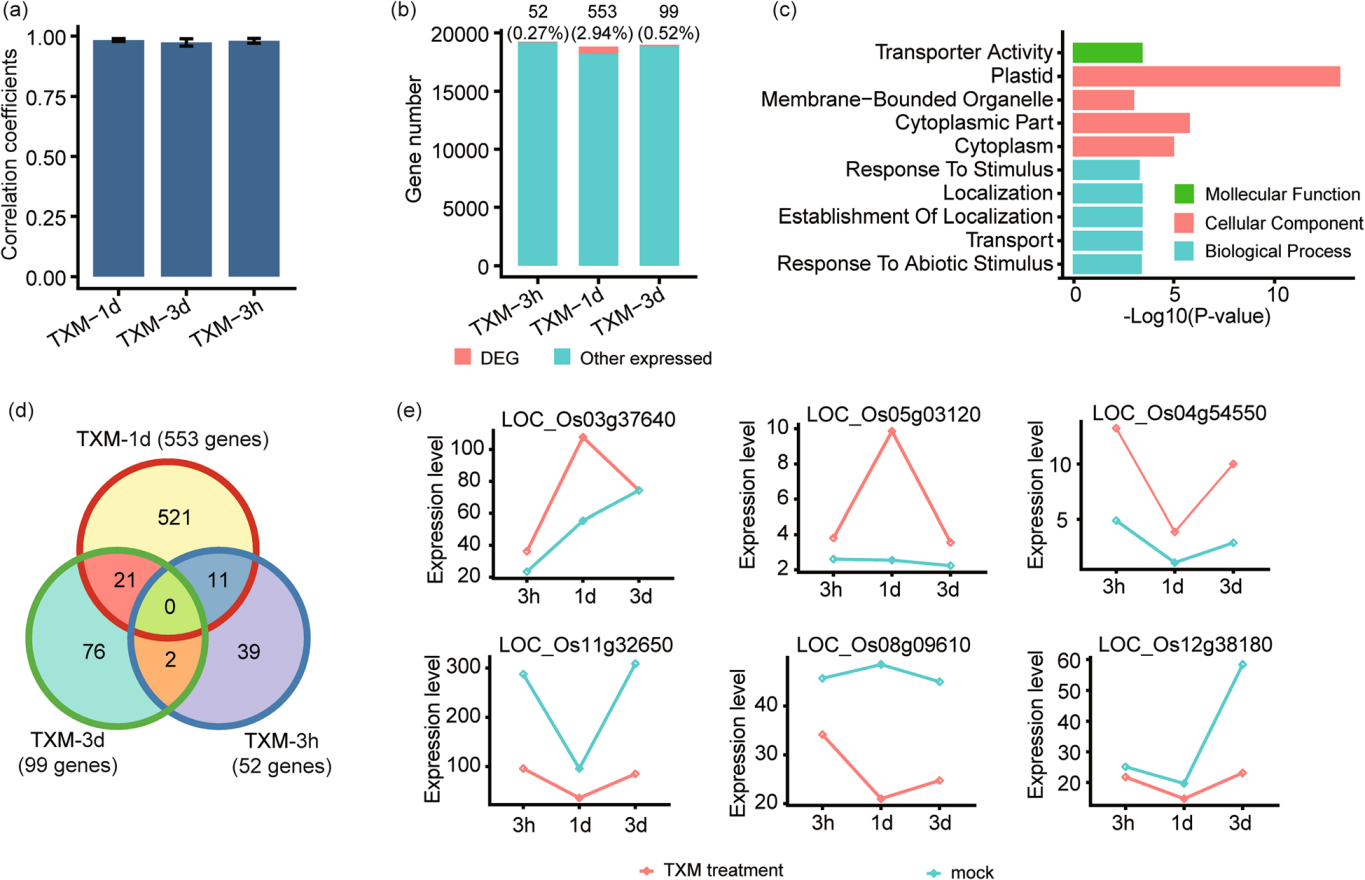
Expression pattern and functional analysis of DEGs in rice inoculated with Thiamethoxam (TXM). **a** Bar graphs show correlation co-efficients (R) of three TXM treatments, i.e., 3 h, 1d, and 3d. The y-axis represents correlation co-efficient of treatments, and x-axis shows pesticide treatments. **b** Bar graphs represent proportionate percentages of DEGs to other expressed genes. The red color in the graph shows the proportion of DEGs to other expressed genes presented in blue color. **c** Overview of GO analysis of the putative DEGs under TXM application. The x-axis represents the negative logarithm of the P-value, and y-axis shows GO terms. **d** Venn diagram is describing total, unique and overlaps among DEGs after treatments with TXM. **e** Expressions of selected DEGs based on high throughput sequencing, under control and TXM treated plants. Expression levels in FPKM of the genes are given on y-axis along with their treatments on x-axis. The first three genes are typical examples of TXM which accumulate more under TXM treatments compared to control, while others are examples of low expressed genes under TXM treatments

TXM has a little more impact on GE level compared to ABM, as a total of 670 DEGs were detected in TXM treated rice. Reliability upon experiment was checked by correlation coefficients (R) which were near to 1 for all treatments (Fig. 2a). To further prevail the effectiveness of TXM, we compared DEGs with other expressed genes in terms of their percentages, and got highest number of DEGs 553 (2.94%) under 1d treatment of TXM, followed by expressions of 99 (0.52%) DEGs under 3d treatment, and 52 (0.27%) DEGs under 3 h treatments (Fig. 2b), adumbrating the fluctuating influence of this pesticide. DEGs were then annotated into functional categories using negative log10 (*P*-value), which illustrated their involvement into transport, localization or response to stimuli GO terms (Fig. 2c). Besides specifically expressed genes, there was a very small proportion overlap in DEGs per time point by TXM (Fig. 2d, Additional file 3). Furthermore, FPKM values of various DEGs with the abiotic stimulus, plastid, and transporter activity have dynamically changed in response to pesticide treatments (Fig. 2e), indicating that the application of TXM can induce some stress responses in rice.

### Identification and characterization of the co-expressed DEGs by two pesticides

**Fig. 3:**
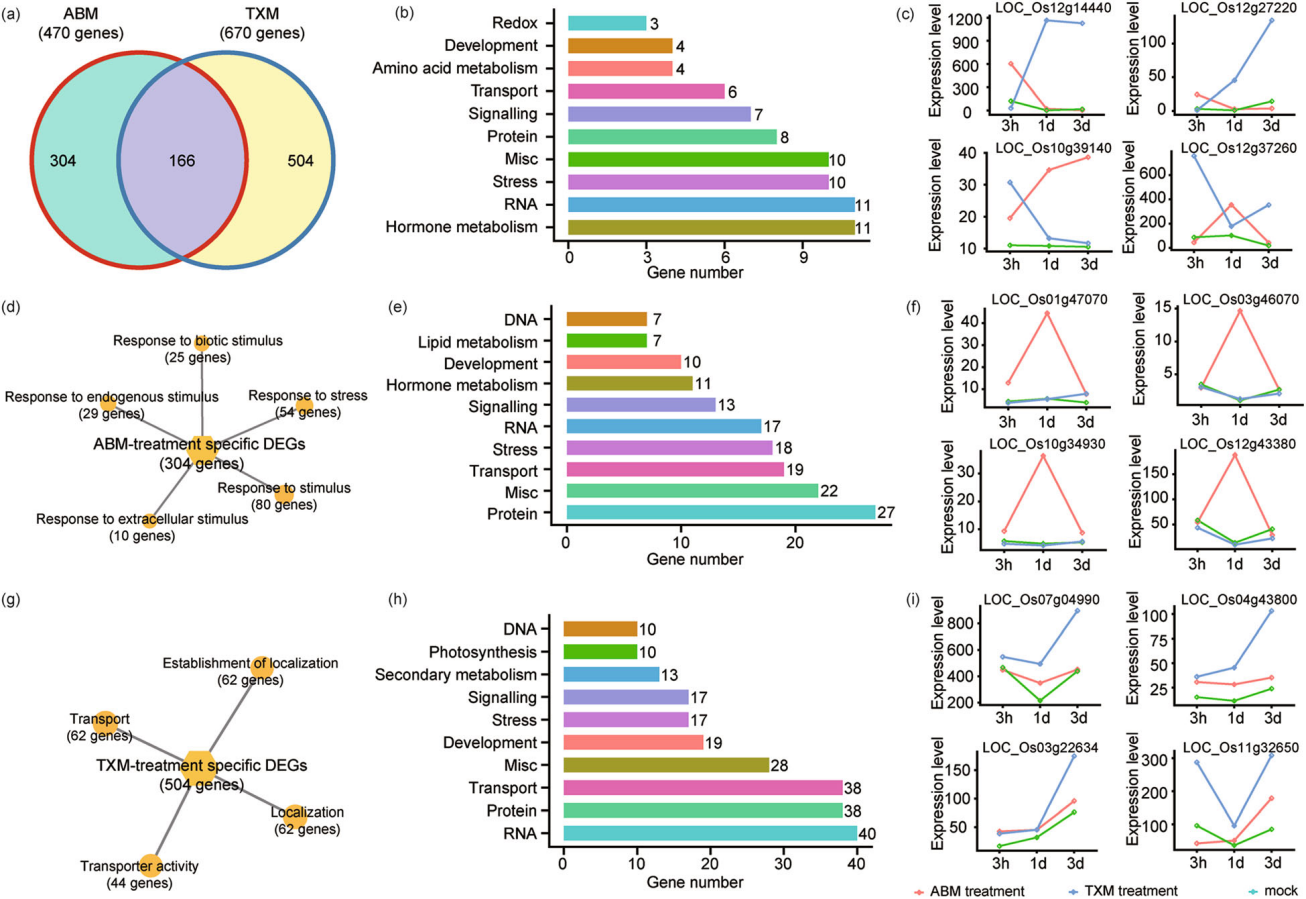
Expression profiles and functional distribution of co-expressed DEGs under ABM and TXM treatments. **a** Comparison of shared and unique DEGs under ABM and TXM treatments in rice. **b** MapMan pathway analysis for all co-expressed DEGs identified between ABM and TXM. The y-axis shows the distribution of genes into different pathways, while x-axis represents a number of genes assumed for each category. **c** The expression level of representative shared DEGs under control, ABM or TXM treatments. FPKM values are specified on y-axis, while x-axis represents treatment time. **d** Enriched GO terms of DEGs annotated in biological processes specific to ABM treatment. **e** Enriched MapMan pathways analyses for all unique DEGs expressed under ABM insecticide application. **f** DEGs specifically responsive to ABM treatments at different time intervals. **g** GO enrichment of TXM special DEGs. **h** Enriched MapMan pathways analyses for all unique DEGs of TXM. **i** Expression profiles of TXM representative DEGs under control, ABM or TXM treatments at three intervals. The expression level of genes is in FPKM, specified on y-axis, while x-axis represents treatment time

DEGs co-expressed among pesticides treatments are of prime importance due to their responses to both pesticides. After examining the individual effects of two pesticides, we scrutinized the co-expressed DEGs, and found 166 shared DEGs expressed under both insecticides treatments (Fig. 3a). To further study the localization and potency of the co-expressed DEGs, we carried out MapMan analysis in detail. These DEGs were mapped into hormone metabolism, RNA, stress, miscellaneous, protein, and signaling pathways with proportionate percentages of 6.62, 6.62, 6.02, 6.02, 4.81 and 3.61%, respectively (Fig. 3b). Notably, we found a significant upregulation of *Os12g27220*, which encodes Spermidine hydroxyl cinnamoyl transferase 1, an enzyme responsible for the biosynthesis of alkaloids, terpenoids, and phenolics (Fig. 3c) [19, 20], proclaiming more synthesis of spermidine may provide plants protection from diseases and pests by using agricultural chemicals.

Next, we examined DEGs specific to each drug by MapMan analysis. We found ABM-specific DEGs involved in several important processes, including response to stimuli, signaling, transport and protein (Fig. 3d, e). Interestingly, we noticed an induced expression level of some DEGs under 1d treatments of ABM compared to control or TXM, but decreased apparently at 3d treatment, indicating no longer effects of ABM on the expressions of these DEGs (Fig. 3f).

Specific DEGs under TXM treatments, by contrast, involved in different localization-related GO terms and cellular processes (Fig. 3g, h). Selected DEGs with unstable expressions indicated that TXM similar to ABM, has limited lasting effects on some GEs in rice (Fig. 3i, Additional file 3). Taken together, these data enlightened the limited roles of the two pesticides in GE regulation.

### Identification of alternative splicing (AS) events in pesticides applied rice

**Fig. 4:**
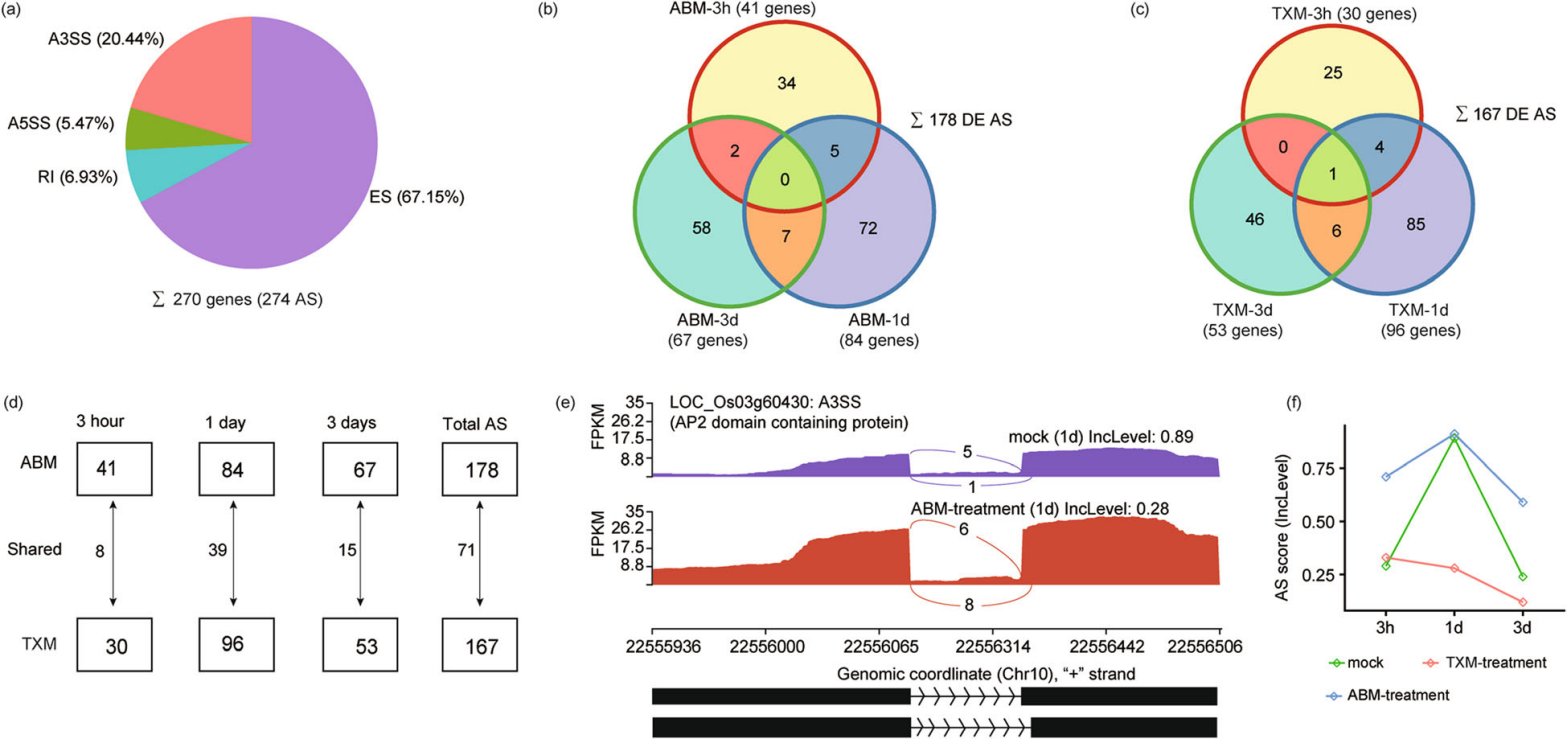
Statistics of differentially expressed alternatively spliced (DE AS) genes under ABM and TXM treatments. **a** Pie chart represents all expressed DEGs (270) with Alternative Splicing (AS) events (274). AS genes along with their percentages are divided into four sub-categories; Exon skipping (ES), Alternative 3’splice site (A3SS), Alternative 5’splice site (A5SS), and Intron retention (IR). **b** Venn diagram represents shared and unique DEGs and their approximate DE AS under ABM treatments. A total number of DE AS (inside) and genes expressed (outside) at each treatment of ABM are specified along with treatment information. **c** Venn diagram represents shared or unique DEGs and their approximate DE AS under TXM treatments. **d** Graphical distribution of DE AS in response to ABM or TXM treatments. Total unique and shared DE AS concerning time are provided in squares or alongside arrows, respectively. **e** An example of A3SS of *Os03g60430*, AP2 domain-containing protein at relatively 1d treatment under control or ABM. Graphical representation of the gene showing AS activity at 3 site under ABM treated samples. **f** AS score (lncLevel) is predicted as an example under control, ABM and TXM treatments at three intervals. The y-axis shows the AS score, the highest is 1, while x-axis demonstrates the three treatments. Graph shows high AS activity of *Os03g60430* under control and TXM at 1d treatment

In addition to be used for DEGs, RNA-seq dataset is also a good resource for AS analysis. Thus, we examined the AS changes affected by pesticides, and acquired approximately 3725 genes undergoing 5779 AS events (Additional file 4). Of them, 270 genes experienced 274 Differentially Expressed Alternative Splicing (DE AS) activity under both pesticides treatments (Fig. 4a and Additional file 5).

We classified total DE AS into four types; i.e., Exon skipping (ES), the most abundant (67.15%) of AS events, followed by Alternative 3′ splice site (A3SS) (20.44%), Intron retention (IR) (6.93%) and Alternative 5′ splice site (A5SS) (5.47%) (Fig. 4a and Additional file 5). We further investigated how AS contributed specifically to either ABM or TXM. For this purpose, we measured 178 DE AS events under ABM and 167 DE AS events under TXM, respectively, consisting of special and shared events among treatments (Fig. 4b, c and Additional file 5). Interestingly, we perceived the highest number of DE AS events under 1d treatments for both pesticides (special or shared), while this number reduced again under 3d treatment as in DEGs (Fig. 4d and Additional file 5), showing a dynamic alternation of AS triggered by these two agricultural chemicals. We predicted an A3SS AS event of *Os03g60430*, a AP2 domain protein-encoding gene, showing high AS activity under ABM compared with control samples under 1d treatment (Fig. 4e). Gene model presented a phenomenon that AS activity has happened in ABM treated samples, avoiding portion from the DEG on its 3′ site (Fig. 4e and f). These results evident that AS alterations could be occurred with pesticides.

**Fig. 5:**
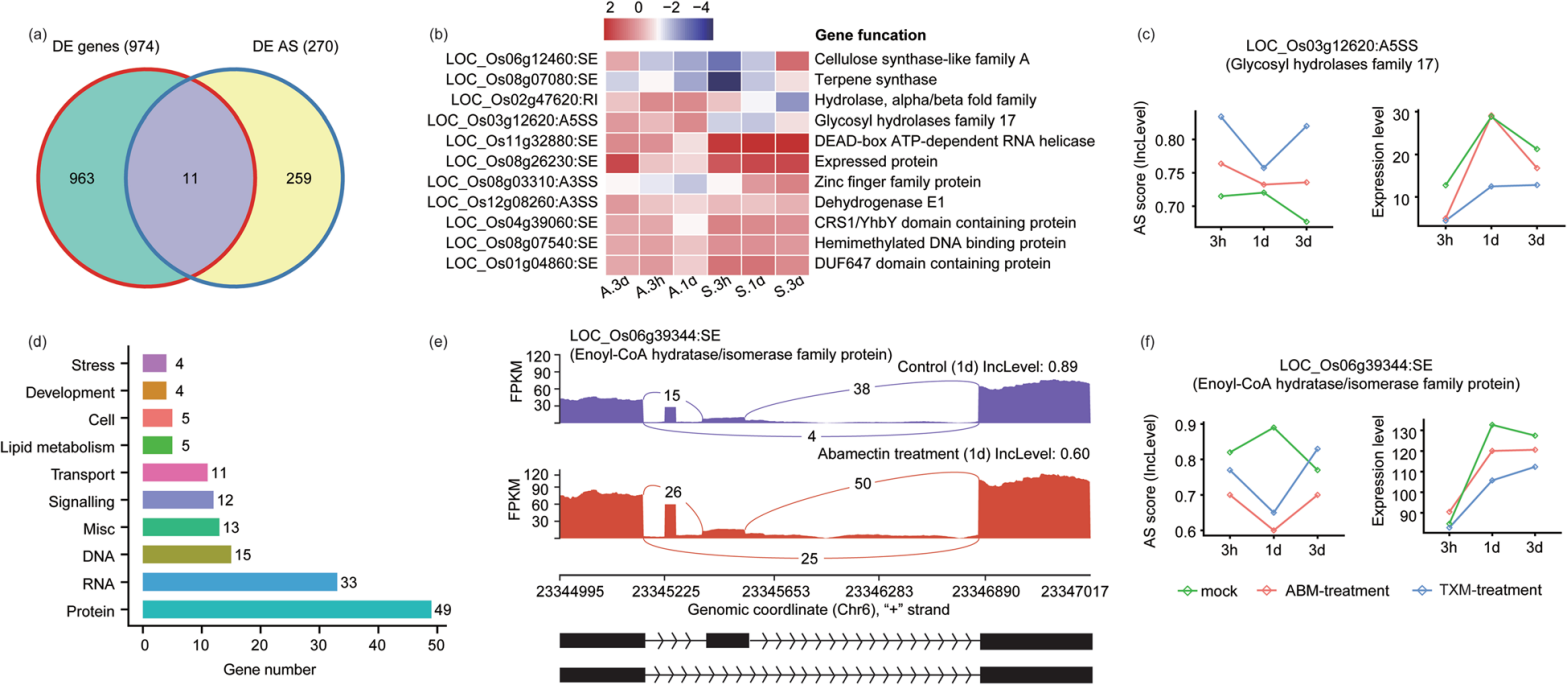
Relative expression and functional distribution of shared DEGs with DE AS under ABM and TXM treatments. **a** Venn diagram represents shared and unique DEGs and DE AS under ABM and TXM treatments. **b** Heat map represents the expression level of selected genes along with their functions and AS activity under insecticides treatments. Transcript levels following insecticides treatments are depicted using FPKM values on a color scale. The spots highlighted in Pink-magenta indicated the DEGs exhibit a significant expression level compared with control after treatments. **c** Expression level of the representative DE AS gene *Os03g12620* under control, ABM and TXM treatments. The left side figure represents the AS score, while right side shows the expression level of AS-mediated gene depicting negative relationship. **d** Enriched MapMan pathways for DE AS events expressed under control, ABM and TXM treatments. The y-axis represents the distribution of genes into different cellular components, and x-axis shows gene numbers indicated in front of each bar. **e** An example of Exon skipping (ES) of *Os06g39344* gene along with its AS score (lnc level) at 1d treatment under control or ABM treatments. Predicted graphical representation of the AS activity can be observed in the form of one exon skipping from the ABM treated samples. **f** Expression profile of representative DE AS gene under control, ABM and TXM treatments at three different intervals. The left side graph represents the AS score of DE AS, while right side shows the expression level of the gene with negative correlation reducing the expression level of the gene under pesticides treatments compared to control samples

Concurrently, we compared DEGs undergoing AS and transcriptional changes at the same time in response to studied pesticides, and got 11 DE AS events (Fig. 5a and b). As an illustration, the AS activity and gene expression of *Os03g12620*, which encodes Glycosyl hydrolases family 17, are oppositely regulated by pesticides (Fig. 5c).

Subsequently, we carried out the distribution of DEGs by DE AS into different pathways using MapMan analysis, and found them enriched in RNA and protein metabolic processes (Fig. 5d). An interesting example is that a gene model of the enzyme, Enoyl-CoA hydratase/isomerase family protein, declared the skipping of one exon under 1d ABM samples, accompanied by an increase of expression under ABM treatment compared to control, exclaiming AS involvement in the GE regulation after a drug spray (Fig. 5e and f). Further research will be interesting to explore the biological significance of DEGs by DE AS in plants under insecticidal environments.

### Characterization of long non-coding RNAs (lncRNAs) in rice under pesticides treatments

**Fig. 6:**
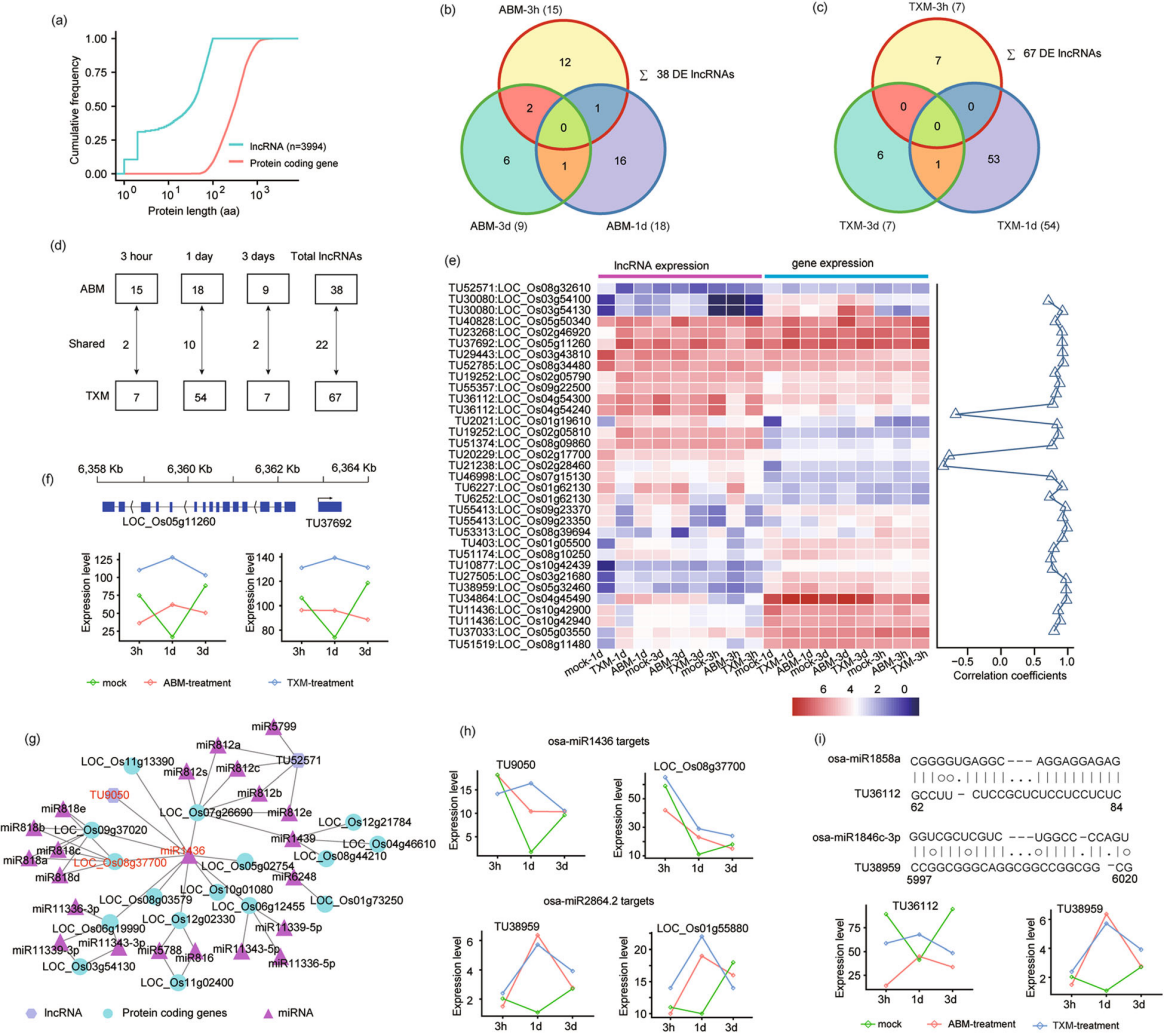
Expression profiles of differentially expressed long non-coding RNAs in rice exposed to two pesticides. **a** Line graph represents a total number of predicted expressed lncRNAs and protein-coding genes (PCG). Predicted length (aa) is shown on x-axis with scale, and cumulative frequency is revealed on y-axis. **b** Venn diagram shows shared and unique DELs perceived under ABM treatments. **c** Venn diagram represents DELs observed at each treatment of TXM. **d** Distribution of total, unique and shared DELs under three treatments responding ABM or TXM. Number of unique and shared lncRNAs are specified in squares or alongside arrows, respectively. **e** Heat map represents the expression level of lncRNAs and their mediated genes in response to the studied pesticides. Color scale indicates FPKM change (blue, low expression level and red, high expression level). Correlation specificity score is presented on the right side of the heat map for lncRNAs and its neighboring genes. Values close to 1 means high correlation (R) of DELs and genes in the vicinity. **f** The expression level of a selected lncRNA and its adjoining gene under ABM and TXM pesticides. Expression levels are assumed on y-axis and treatment time is denoted by x-axis. As an example, lncRNA *TU37692* positive correlates *Os05g11260*. Graphical representation of the gene and lncRNA is shown above graph representing the position of gene and lncRNA. **g** Predicted interaction network of miRNAs, lncRNAs, and PCGs. Circles show PCGs, triangles represent miRNAs and hexagonal structures indicate lncRNAs. Osa-miR1436 is specified as an example, targeting lncRNA *TU9050* and a gene *Os08g37700* highlighted in the red color. **h** Expression levels of two lncRNAs-mediated genes targeted by miRNAs are specified. The y-axis represents expression levels of lncRNAs and PCG and x-axis shows treatments of two pesticides. Osa-miR1436 and Osa-miR2864.2 target lncRNAs and PCGs positively regulating their expression levels under drugs treatments. **i** Predicted base pair interaction between two miRNAs and their targeted lncRNAs. Line graphs show high expression level of lncRNAs under pesticide treatments compared with control

LncRNA has been implicated playing a critical role in coding gene expressions. To predict lncRNAs in our transcriptomic dataset, we analyzed the assembled and filtered transcripts procuring approximately 3994 unique lncRNAs under two pesticides treatments, with 83 differentially expressed lncRNAs [21] among them (Fig. 6a). These differentially expressed lncRNAs (DELs) ranged in length between 270 and 6317 bp, and the most abundant length was 300–500 bp (Fig. 6a, Additional file 6). Furthermore, we distributed DELs individually to ABM and TXM treatments, and found the maximum number of DELs under TXM, consistent with the trend of DEGs, indicating a broader spectrum characteristics of TXM than ABM (Fig. 6b and c). We also examined co-expressed DELs among pesticides treatments and observed a higher number of DELs under 1d treatments (Fig. 6d), submitting a dynamic change of lncRNAs similar to DE AS in pesticides treated rice.

Previous studies have shown that lncRNAs could regulate the expressions of their neighboring protein-coding genes (PCGs) [22–24]. Therefore, we performed hierarchical clustering of the DELs, in which most of the DELs have positively regulated expressions of their neighboring genes (Fig. 6e). As an example, it is obvious that lncRNA *TU37692* is positively regulating the expression of its neighboring Polysaccharide-K gene *Os05g11260* (Fig. 6f).

A major theme involves, is the regulatory role of lncRNAs, which acts as a miRNA “sponge” to trigger the expression of PCGs [25–27]. To predict miRNA, lncRNA and coding genes interactions, we used Cytoscape (http://www.cytoscape.org/) and constructed the putative interactive network of miRNAs targeting their presumed lncRNAs and PCGs. Both, lncRNA *TU9050A* and *Os08g37700* are predicted as miR1436 targets; we observed that they could be simultaneously induced by the two pesticides, suggesting that lncRNA *TU9050A* may block miR1436 activity to accelerate *Os08g37700* transcription (Fig. 6g, h and Additional file 7). Likewise, *Os01g55880*, a target of miR2864.2, could be more induced through the sponge activity of lncRNA *TU38959* under drugs treatments (Fig. 6h and Additional file 8). Nevertheless, we found that *TU36112*, the non-coding target of miR1858, and *TU389592*, the non-coding target of miR1846c-3p, could be dynamically changed, whereas the coding targets of miR1858 and miR1846c-3p were not profoundly altered by pesticide treatments. Thus, whether these two DELs can function as a miRNA target mimicry is unknown and should be further determined (Fig. 6i). Together, this predicted evidence suggest that lncRNAs might be involved in the fluctuating GE of DEGs through regulation of miRNA activities in response to pesticides.

### Transposable elements (TEs) are involved in the alteration of gene expressions in the locale

**Fig. 7:**
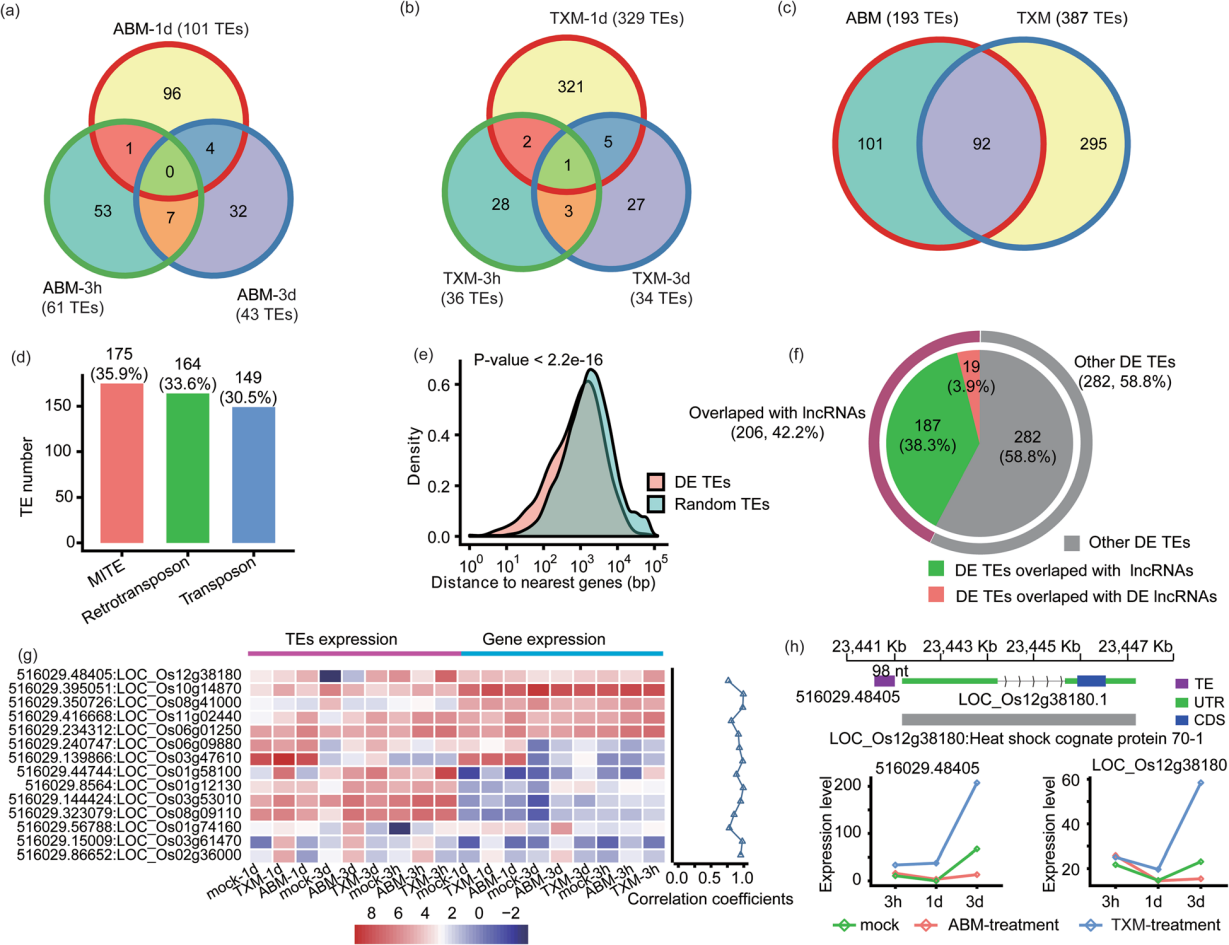
Classification and expression profile of Transposable Elements (TEs) under ABM or TXM treatments. **a** Venn diagram represents shared, and unique DE TEs observed under three treatments of ABM. Total, unique (outside) and shared (inside) DE TEs are specified. **b** Shared and unique DE TEs perceived under different treatments of TXM. **c** Venn diagram depicts special, and co-expressed DE TEs expressed under ABM and TXM applications. **d** Bar graphs represent distribution of total DE TEs into three classes, i.e., Transposons 149, Retrotransposons 164 and Miniature Inverted-repeat TEs (MITE) 175 on the basis of their function. The y-axis represents number of expected DE TEs along with their percentages at the top of each bar and their classification is shown on x-axis. **e** Graph presents density of significant DE TEs and random TEs. The y-axis represents density of DE TEs and TEs (maximum value is 1) while x-axis shows distance to nearest genes (scale given in bp). *P*-value of significance is shown on the top of y-axis. **f** Pie chart represents proportions of DE TEs under pesticides treatments and interaction of DE TEs with DELs or lncRNAs. **g** Heat map shows FPKM values of TEs and adjoining genes in response to the studied pesticides. Color scale indicates expression level of TEs and their neighboring genes. Line graph on the right side of heat map represent correlation co-efficient (R) of TEs and DEGs. Most of the TEs positively correlates their neighboring genes. **h** Example of TE affecting expression level of its neighboring gene. Map shows graphical representation of TE *48405* and HS cognate protein 70–1 gene along with UTR and CDS regions indicating positive correlation of the TE expression and GE

TE insertion is another significant contributor of gene expressions in plants other than lncRNAs. In RNA-seq dataset, TE transcriptions can easily be examined as an asset. So we examined TEs in genes having transcriptional changes in response to pesticides treatments. Firstly, we analyzed DE TEs individually for two pesticides and got 193 and 387 DE TEs under ABM and TXM treatments, respectively (Fig. 7a, b and c). Intriguingly, DE TEs were higher in TXM treatments as that of DE AS, DEGs as well as DELs, further illustrating TXM has stronger effects than ABM on rice. Ninety-two co-expressed DE TEs were ascertained responsive to both pesticides (Fig. 7c, Additional file 9). Furthermore, we classified DE TEs on the basis of their functions and observed that the maximum number of DE TEs are in the class Miniature Inverted-repeat Transposable Elements 175 (35.9%), followed by retrotransposon 164 (33.6%) and transposons 149 (30.5%) (Fig. 7d).

The density of TEs to the nearest genes can result in impacts on transposition conversely affecting transcription. Therefore, we measured the density of DE TEs and TEs to the nearest genes indicated by a peak (*P*-value < 2.2e-16) and found that DE TEs were closer to genes compared to random TEs (Fig. 7e). These results indicated that DE TEs have a higher ability to regulate the nearby genes.

Besides coding regions, GE levels can also be impacted by TE insertions into non-coding regions. We found that more than 42% of DE TEs were overlapped with lncRNAs in our RNA-seq datasets, confirming the phenomenon that GE regulation by TEs can be the result of its insertion into either coding or non-coding DNA zones (Fig. 7f). Furthermore, hierarchical interaction showed that most of the DE TEs were involved in positively regulating their mediated GE levels, for instance, TE-48405 on rice chromosome 12 near to the TSS of the *Os12g38180*, a gene encoding Heat shock cognate 70–1, positively regulated its expression pattern in the vicinity by ABM and TXM treatments (Fig. 7g, h and Additional file 9). The above results suggested that TEs are the crucial means to alter gene transcriptions, playing critical roles in stress responses and defense mechanisms.

## Discussion

As a plant development hormone, auxins play critical roles in plant growth and reproduction. In the present study, we found that ABM regulates many genes with potential functions in the auxin response, including one auxin efflux carrier gene *Os01g69070*, three auxin-repressed genes *Os03g22270*, *Os11g44810*, and *Os08g35190,* and two auxin response SAUR genes *Os06g45970, Os02g07110,* suggesting that ABM may influence the auxin signaling in rice (Fig. 1e). Under stressful conditions, plants would produce a high level of anthocyanins to increase yield and antioxidant capacity [28]. We found that the expression of *Os04g54550* (*AT5MAT*) encoding an enzyme that prevents the degradation of anthocyanins, can be induced considerably by TXM (Fig. 2e). Hence, upregulation of *AT5MAT* may be an adaptive strategy of plants to cope with the toxic effects of insecticide [29]. Heat Shock Proteins (HSPs) are involved in the regulation of specific substrate proteins in stressful conditions especially under high temperature [30]. Under TXM application, some HSPs were also inhibited (Fig. 2e), showing the possibility that disruption of thermal morphogenesis by the farm chemical is an alternative approach to trade off the physiological and defense responses in plants.

Thaumatins are pathogenesis-related (PR) proteins, which are induced by various agents ranging from ethylene to pathogens in plants [31]. Therefore, the identification and functional characterization of thaumatins were pretty interesting. We found that *Os03g46070* and *Os12g43380*, two genes involved in thaumatins biogenesis were increased under ABM treatments (Fig. 3f), indicating thaumatins may play coordinated roles in defense against masticatory insects. Nonetheless, salicylic acid (SA) and jasmonic acid (JA), two phytohormones play roles in resistance to pathogens by inducing the production of pathogenesis-related proteins and are further involved in the systematic acquired resistance of plants where a pathogenic attack on one part of the plant induces resistance in other parts [32, 33]. Here, two genes were alternatively regulated for SA production inducing the expressions of *Os05g01140,* while decrease in the expressions of *Os11g15040* were illustrated under two pesticides treatments. Furthermore, *Os09g02710, Os08g39850, Os12g37260 and Os03g28940,* four genes involved in JA synthesis were increased during pesticides application (Additional file 10). These results suggested that application of studied pesticides reprogramed different metabolic pathways by modulating the expressions of critical DEGs in rice.

*Os11g32880*, a DEAD-box ATP-dependent RNA helicase (DBH) encoding gene, was subjected to AS and transcriptionally regulated by TXM treatments (Fig. 5b). It is noteworthy that there are a lot of redundant helicases in rice, which might be the result of either tandem or segmental duplications [34], thus whether AS of *Os11g32880* can regulate other helicases by pesticides remains unknown, since AS events sometimes exert dominate-negative effects on gene functions [35]. As a defense signal to IPs, terpene synthase, which is involved in secondary metabolism and biosynthesis of phytoalexins, was negatively regulated by high ES under both ABM and TXM treatments [36], suggesting that pesticide can regulate these defensive genes at post-transcriptional level to enhance plant biotic resistance. Taken together, the subsequent induction in expression of many stress-responsive genes indicated that AS might be involved to persuade defense mechanism of rice in response to pesticides.

In a meanwhile, this study was motivated by our interest in the spatio-insecticidal incentives of lncRNAs, and exploring its potential biological roles in GE modulation in rice treated with pesticides. Interestingly, we found an elevated number of DELs at 1d treatment under both pesticides, which evidenced that the increased number of DEGs might be due to the involvement of adjacent lncRNAs (Fig. 6b and c). Add-on to this, most of the lncRNA-mediated genes belong to either metabolic, stress-responsive pathways or involved in defense mechanisms (Fig. 6e). Through the potential to create new transcripts, premature termination, or novel promoters, TEs can influence the expression patterns of plants genes [37, 38]. Here, we have evaluated a variety of TEs that can interact with neighboring genes to shape transcripts and regulate their expression levels. For instance, C-glycosylflavone coding gene, *Os06g01250*, is critical for defense against plant-eating insects [39–41], was positively regulated by TE-234312 in rice under both chemicals, determining its roles in defense response probably via TE mediated regulations (Fig. 7g). NB-ARC domain is found in bacteria and eukaryotes and shared by plant resistance (R) proteins proposed to be involved in pathogen recognition [42]. Interestingly, we found *Os08g09110*; an NB-ARC R gene was negatively regulated by TE-323079 under both pesticides treatments, indicating agricultural drugs may affect R gene expressions indirectly by regulating TEs.

## Conclusion

To conclude, this study provides the first systematic analysis of gene expressions responsive to two commercial pesticides in rice, which can be further applied for other crops and alternative agricultural chemicals. Although we detected several DE AS, DELs and DE TEs alterations accompanied with host GEs, our study overall depicted that recommended doses of ABM and TXM pesticides are effective in controlling IPs and likely to have no apparently harmful effects on plant growth and physiological performance in rice.

## Methods

### Plant materials

Rice (*Oryza sativa*), cv. Xidao No.1, spp. japonica WT was used in the study. Seeds were soaked in deionized water overnight at 28^ο^C and then transferred to a net floating on a 0.5 mM CaCl_2_ solution. After 7 days, seedlings were transplanted into soil in a greenhouse (approximately 28/25^ο^C day/night, 75 ± 85% relative humidity) and grown for 6 weeks. Pesticides ABM and TXM were applied at the jointing stage (6 weeks) using the recommended rate; i.e.,1.81 gh^− 1^ of ABM and 3 g ai 100 l^− 1^, (300 l h^− 1^) of TXM [14]. Pesticides were applied at 8 o’clock in the morning and samples (ABM, TXM and control) were collected at three intervals; i.e., 3 h, 1 day and 3 days after pesticides spray. All nine samples (each one containing three biological replicates) were immediately frozen in liquid nitrogen and stored at − 80 ^ο^C for RNA extraction.

### RNA extraction and Illumina sequencing

Three biological replicates were used for all RNA-Seq experiments sampled from control and pesticide-treated plants. Total RNA was extracted from each sample using TRIzol Reagent (Invitrogen)/RNeasy Mini Kit (Qiagen). Total RNA was quantified and qualified by Agilent 2100 Bio-analyzer (Agilent Technologies, Palo Alto, CA, USA), Nano Drop (Thermo Fisher Scientific Inc.) and 1% agarose gel. RNA with RIN value above 7 was used for further sequencing library construction. RNA-sequencing was performed on an Illumina HiSeq2500 platform (PE 150 bp) at Novogene Company (Beijing, China). The detailed information for RNA-seq data is listed in supporting information Additional file 1. All the RNA-seq data have been deposited in NCBI database with accession number PRJNA532802.

### Analysis of RNA-seq data

Rice genomic sequence (*O. sativa*_323_v7.0) and the corresponding annotation was retrieved from the Phytozome database (Version 12.0) [43]. TEs were extracted from the rice repeat annotation file (Osativa_323_v7.0.repeatmasked_assembly_v7.0.gff3). Only TEs derived from the intergenic region remained for further analysis. After filtering adaptor and low quality reads with FASTX-Toolkit (version 0.0.14), all clean data for each sample were separately aligned to the reference genome using Hisat2 with the following parameters: “--end-to-end, --known-splice site-infile” [44]. For each sample, unique mapped reads were assembled into putative transcripts based on a reference-guided assembly strategy using StringTie (version 1.3.3b) [44]. The meta-assembly tool TACO (version 0.7.3) was used to merge putative transcripts from each sample into a unified set of transcripts [45], which was then compared to the reference gene GTF file using gffcompare (version 0.10.1) [46].

Based on transcript classification codes, “u” (unknown) intergenic transcripts were regarded as novel gene loci and used to lncRNA identification. Five steps were adopted to identify bona fide lncRNAs as previously described [47]: (1) transcripts should be with length ≥ 200 bp and detected in more than 3 samples; (2) transcripts derived from rRNA and tRNA were removed (cutoff E-value0.001); (3) transcripts encoding proteins and protein-coding domains were removed by searched against the Swiss-Prot and Pfam databases (cutoff E-value 0.001); (4) OrfPredictor was applied to predict ORFs and transcripts that encode more than 100 amino acid was removed [48]. (5) transcripts were removed that did not pass the protein-coding-score test using Coding-Non-Coding Index (CNCI) [49] and Coding Potential Calculator (CPC) software [50].

Resulting lncRNA transcripts and known transcripts, intergenic TEs were then merged into non-redundant transcripts, which were further quantified by StringTie for each sample [46]. Differential expression analysis for each sequenced library was performed using ballgown [44]. The corrected *P* value of 0.05 and abs |log2 (Fold change)| of 1 were set as the threshold for significant differential expression. Singular Enrichment Analysis from AgriGO was performed to identify significantly enriched GO terms in the gene list out of the background of the reference gene list [51]. GO terms pathways with false discovery rate (q-value) < 0.05 were considered as significantly altered.

All putative AS events were extracted from the rice transcript GTF file using rMATS [52]. Expressed AS events were identified by filtered below three samples with total IJC + SJC < 30. Differentially AS events between control and each pesticide-treated condition were identified using rMATS with a stringent threshold: FDR ≤ 0.05, Delta PSI (ΔPSI) ≥10%.

### Prediction of miRNA, lncRNA and coding genes network

Pearson correlation was employed to explore the expression relationship between lncRNAs and their neighboring genes (≤ 10 Kb). Mature miRNAs for rice were retrieved from the miRBase database (Release 21.0). To identify lncRNAs and genes as putative miRNA targets and mimics, the TAPIR tool was used with the default settings [53]. The relationship between miRNAs, lncRNAs, and genes were used to construct the interaction networks with Cytoscape software (version 3.5.1) [54].

### Validation of DEGs by RT-qPCR

To validate RNA-seq, we selected six genes based on their expressions from DEGs and tested their expressions using qRT-PCR. RNA used earlier for RNA-seq samples were reversely transcribed using reverse transcriptase cDNA synthesis kit (GoScript™ Reverse Transcription System, Promega, Beijing, China). qRT-PCR analyses of genes were performed as previously described [55]. Real-time qPCR was done in triplicates on Step-One Plus real-time PCR system (ABI) with the Power Up SYBR Master Mix (ABI) with three biological replicates. The following cycling conditions were used for Real-time qPCR: 2 min at 95 °C, 40 cycles of 10 s at 95 °C and 40 s at 65 °C, and a final step for melting curve determination (15 s at 95 °C, 1 min at 60 °C and 15 s at 95 °C). ACTIN was used as an internal control. Gene expression was calculated using the 2^-ΔΔCt^ method. Primers used for gene expression analysis are listed in Additional file 11.

## Supplementary information

**Additional file 1.** Mapping statistics of mock and pesticides applied three treatments. (A-Abamectin; T-Thiamethoxam)

**Additional file 2.** Confirmation of the expression patterns of DEGs using real-time quantitative polymerase chain reaction (RT-qPCR)

**Additional file 3.** DEGs identified by RNA-seq analysis under two pesticides treatments expressed in FPKM

**Additional file 4.** Pie chart represents all expressed genes and total AS activities observed in the study. All AS activities were divided into five types; i.e., Exon skipping (ES), Alternative 3′ splice site (A3SS), Alternative 5′ splice site (A5SS), Mutually exclusive exon (MXE) and Intron retention (IR). Percentage of each type is also acknowledged

**Additional file 5.** DEGs identified executing AS events under two pesticides treatments

**Additional file 6.** DELs identified under two pesticides treatments expressed in FPKM

**Additional file 7.** Predicted interaction network of miRNAs, lncRNAs, and PCGs. Circles show PCGs, triangles represent miRNAs, and hexagonal structures indicate lncRNAs

**Additional file 8.** Differentially expressed genes targeted by miRNAs under pesticides treatments

**Additional file 9.** DE TEs identified in rice under two pesticides treatments

**Additional file 10.** DEGs related to salicylic acid and jasmonic acid synthesis for rice under two pesticides treatments

**Additional file 11.** List of Primers used for validation of six auxin responsive genes

## Data Availability

All the raw reads produced in this study have been deposited in NCBI database with accession number PRJNA532802.

## Abbreviations

A3SS: Alternative 3′ Splice Site
A5SS: Alternative 5′ Splice Site
ABM: Abamectin
AS: Alternative Splicing
CNCI: Coding-Non-Coding Index
CPC: Coding Potential Calculator
DE AS: Differetially Expressed Alternatively Spliced
DE TEs: Differentially Expressed Transposable Elements
DEGs: Differentially Expressed Genes
DELs: Differentially Expressed Long Non-coding RNAs
ES: Exon Skipping
FPKM: Fragments Per Kilobase of exon per Million reads
IR: Intron Retention
lncRNA: Long Non-conding Ribonucleic Acid
miRNAs: MicroRNAs
MXE: Mutually Exclusive Exon
PCGs: Protein Coding Genes
RNA-seq: RNA Sequencing
TEs: Transposable Elements
TXM: Thiamethoxam
