# Supplementary figures and images for: Pesticide application has little influence on coding and non-coding gene expressions in rice
Source: BMC Genomics. 2019 Dec 23;20:1009. doi: 10.1186/s12864-019-6381-y (PMC6927115; doi:10.1186/s12864-019-6381-y)

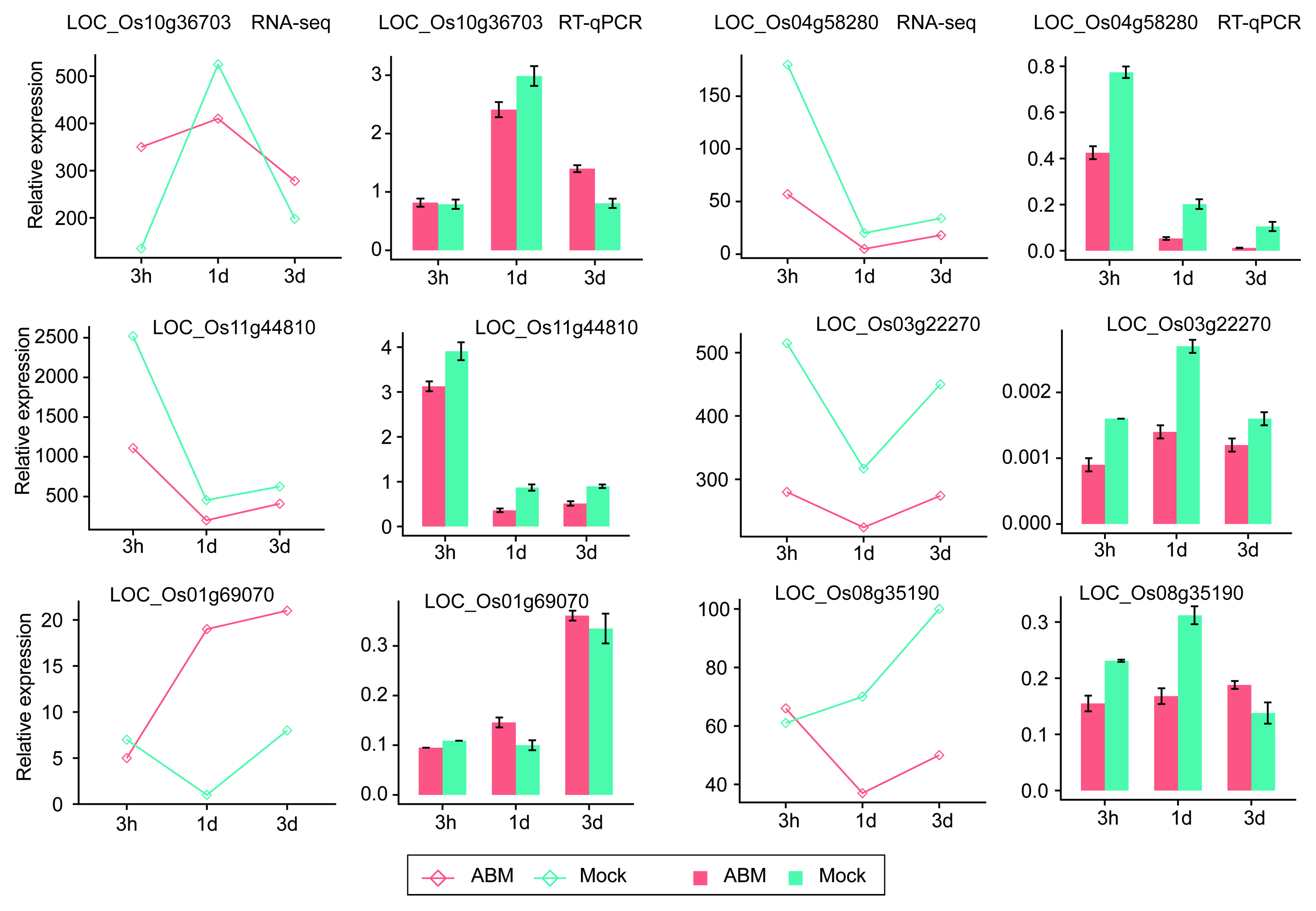

Supplement: Supplementary file 2 — Additional file 2. Confirmation of the expression patterns of DEGs using real-time quantitative polymerase chain reaction (RT-qPCR) [file 12864_2019_6381_MOESM2_ESM.tif]

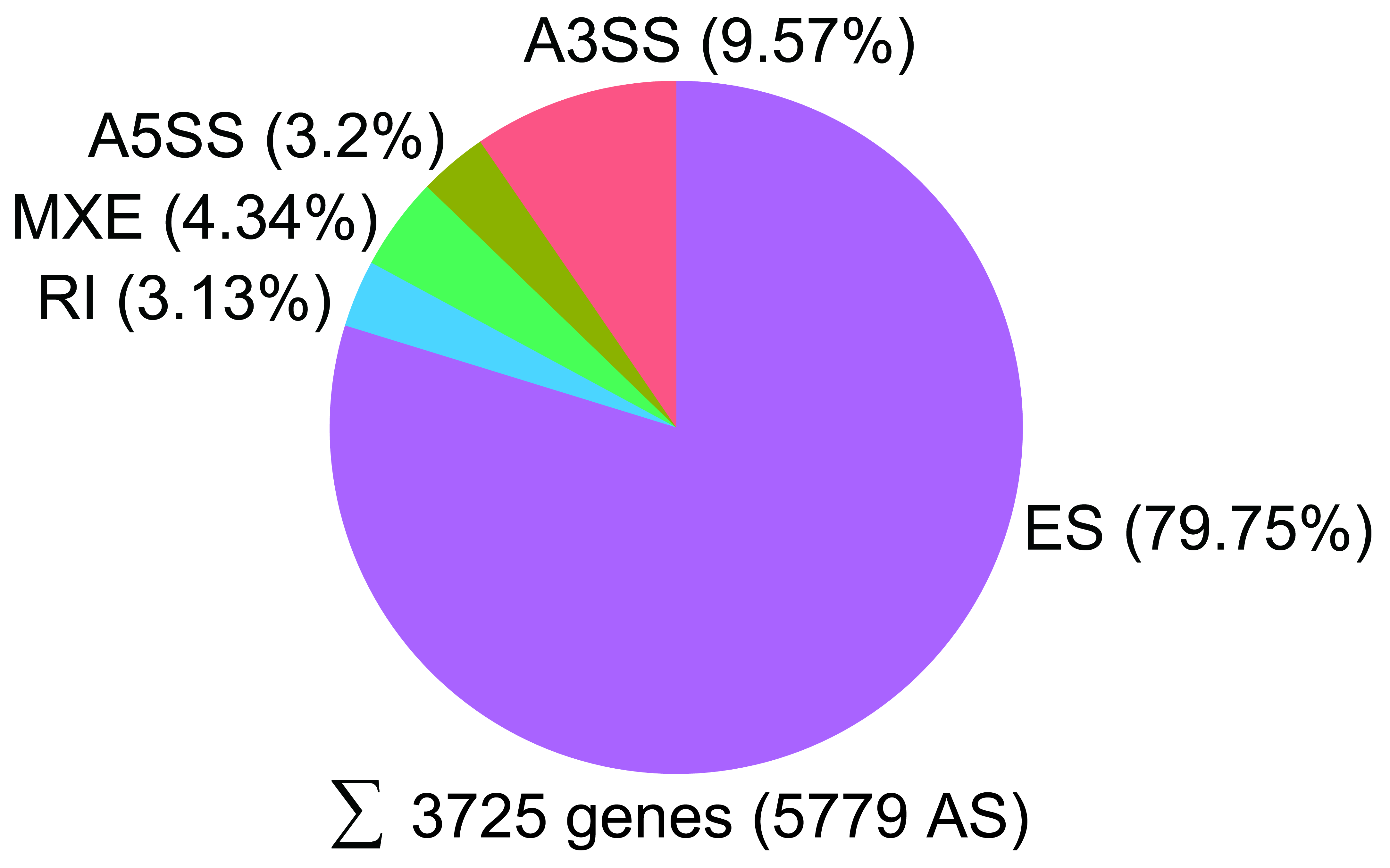

Supplement: Supplementary file 4 — Additional file 4. Pie chart represents all expressed genes and total AS activities observed in the study. All AS activities were divided into five types; i.e., Exon skipping (ES), Alternative 3′ splice site (A3SS), Alternative 5′ splice site (A5SS), Mutually exclusive exon (MXE) and Intron retention (IR). Percentage of each type is also acknowledged [file 12864_2019_6381_MOESM4_ESM.tif]

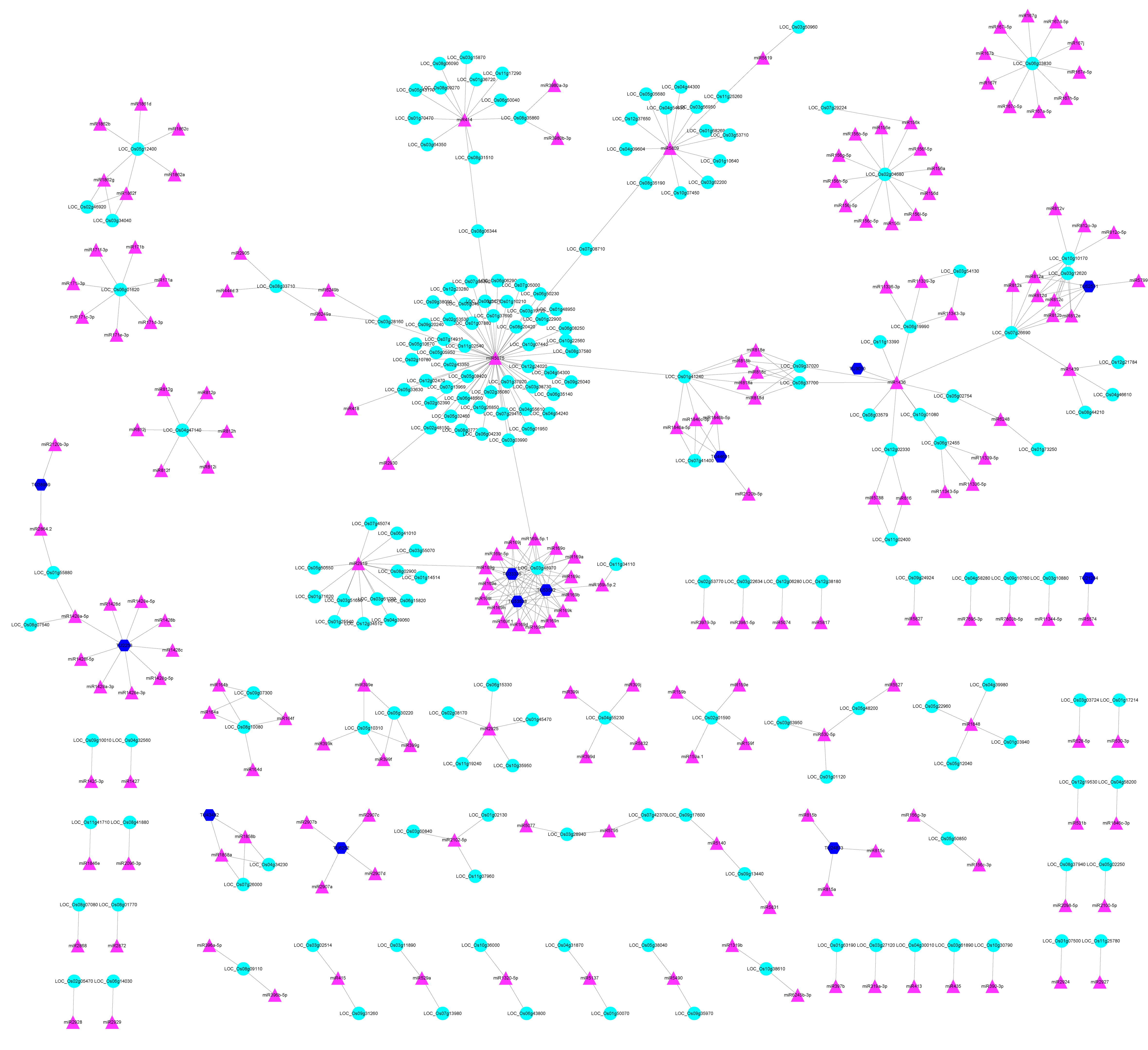

Supplement: Supplementary file 7 — Additional file 7. Predicted interaction network of miRNAs, lncRNAs, and PCGs. Circles show PCGs, triangles represent miRNAs, and hexagonal structures indicate lncRNAs [file 12864_2019_6381_MOESM7_ESM.tif]

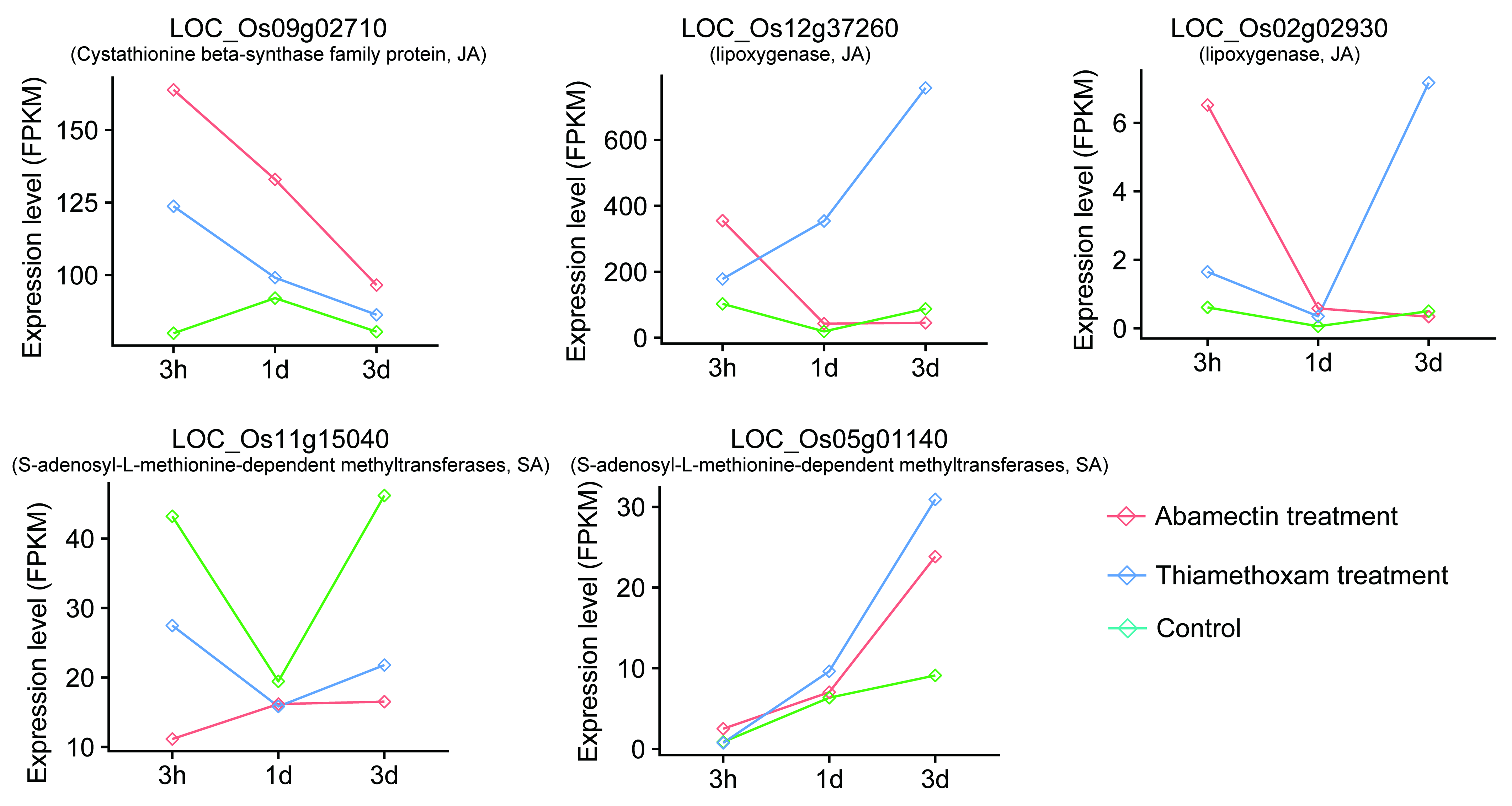

Supplement: Supplementary file 11 — Additional file 11. List of Primers used for validation of six auxin responsive genes [file 12864_2019_6381_MOESM11_ESM.tif]
